# Exploring the Mechanism of Pathological Gaming in Adolescents: Focused on the Mediation Paths and Latent Group Comparison

**DOI:** 10.3389/fpsyg.2021.756328

**Published:** 2022-01-12

**Authors:** Hyeon Gyu Jeon, Eui Jun Jeong, Sung Je Lee, Jeong Ae Kim

**Affiliations:** ^1^Department of Digital Culture and Contents, Konkuk University, Seoul, South Korea; ^2^Department of Humanities Counseling and Therapy, Konkuk University, Seoul, South Korea

**Keywords:** pathological gaming, academic stress, self-control, longitudinal study, aggression, LCA

## Abstract

Pathological gaming among adolescents has been reported to hamper the achievement of a balanced life and to threaten the development of social competencies. Despite the increasing social concerns on the adolescent users, however, the mechanism of gaming behavior of adolescents has not been sufficiently examined. This study explored the mechanism of pathological gaming among adolescents from 3-year longitudinal data of 778 Korean adolescent gamers, by analyzing the effects of negative affects (i.e., anxiety, loneliness, and academic stress) on the degree of pathological gaming through the mediation variables (i.e., aggression and self-control) based on the stimulus-organism-response (S-O-R) framework. Latent class analysis (LCA) was used to uncover potential risk groups, and through partial least squares-structural equation modeling (PLS-SEM) analysis, the mediation pathways to pathological gaming were compared between the risk group and the non-risk group. The results highlighted the key role of academic stress on the degree of pathological gaming. In the entire group, academic stress primarily increased pathological gaming through self-control. The mediation path of self-control was the most influential result in the risk group. Aggression was the key mediator between loneliness and pathological gaming in the non-risk group. The theoretical and practical implications of the results were discussed.

## Introduction

Pathological gaming hinders the achievement of a balanced life by weakening self-control over game use and thereby hampering the development of academic and work competencies (World Health Organization, 2018). Adolescents are particularly vulnerable to pathological gaming because they are still developing their ability to master themselves (Kuss et al., 2013; Lopez-Fernandez et al., 2014; Torres-Rodríguez et al., 2019). Thus, adolescents have a high risk of experiencing problems due to addictive symptoms such as imperfect identity integration, unpredictable behavior and its consequences, and neurobiological immaturity (Steinhausen and Metzke, 2001; Kuss et al., 2013; Torres-Rodríguez et al., 2018, 2019). With recent surveys showing that 75% of male adolescents in highly developed Western countries immerse themselves in game use weekly (Islam et al., 2020) and 85% of Korean adolescents use games about 90 min daily (Cui et al., 2018; Korean Creative Content Agency, 2020), game use among adolescents is approaching danger and is threatening future social balance (Hawi et al., 2018). In order to understand the increase in the use of addictive games among adolescents and their effects, it is necessary to understand the mechanisms of adolescents’ gaming behavior.

Negative affects such as anxiety attract individuals to pathological gaming by cultivating evasive coping attitudes such as denial of the problem, avoidance of problem solving, and pursuit of immediate compensation (Caplan, 2002). This evasive coping mechanism can breed such a vicious cycle of sequential repetition that the individual becomes excessively immersed in the game (Petry et al., 2014; Paulus et al., 2018). When access to the game is restricted, further negative affects may be experienced, such as irritability and sadness (King et al., 2020). However, psychological factors of pathological gaming among adolescents, such as anxiety, loneliness, and stress, are not yet sufficiently understood (Mehroof and Griffiths, 2010; Cheng et al., 2018; Ohannessian, 2018). A more in-depth and multi-faceted study on the relationship between pathological gaming among adolescents, their negative affects, and their not yet fully developed ability to control their emotions compared to adults is required to arrest pathological gaming among adolescents (Ahmed et al., 2015).

Coping with pathological gaming has been found to require control and suppression of affects, especially negative affects (Yen et al., 2018). Control of negative affects implies interventions to prevent negative affects from directly causing pathological gaming. This idea suggests that negative affects can be mediated by organic operations before reaching game addiction, which is inferred from the stimulus-organism-response (S-O-R) framework (Arora, 1982; Islam and Rahman, 2017). The S-O-R model will help us understand the patterns within stimulus-organism operations of individual behaviors, such as pathological gaming. Studies on pathological use of the internet and games have confirmed that organic factors such as aggression (Jeong et al., 2017) and self-control (Song and Park, 2019; Mills and Allen, 2020) significantly mediate the negative affects pathological use connection. However, the results of such studies had limited significant implications on pathological gaming among adolescents because the study subjects were adults or few affects, such as depression, were dealt with. In order to understand the pathological gaming of adolescents, a comprehensive review of the extended model would be appropriate.

Deriving robust results by revealing the latent class in a sample is an important and significant challenge, as it can resolve the validity threats and biases of the conclusion. However, latent class analysis (LCA) has been rarely used in behavioral addiction studies and even more rarely used in longitudinal studies (see Table 1). Moreover, researchers have attempted to divide the data into several subgroups through a monotonous method that uses observed heterogeneity in an *a priori* context, such as demographic variables (gender, age, region, etc.) or psychological variables (personality, neurosis, etc.). However, this traditional approach is effective only when it can clearly identify the observed heterogeneity variables; and it has the limitation of not reflecting at all the peculiarity of a model using research variables. Therefore, the robustness of research results must be ensured through non-empirical and non-predictive approaches such as LCA, which reveals the unobserved heterogeneity inherent in the data itself (Rigdon et al., 2010; Becker et al., 2013; Sarstedt et al., 2020).

**Table 1:** Prior research using latent class analysis (LCA) in behavioral addiction.

Author(s)	Year	Domain	Key variables	Subjects	Data type
Tullett-Prado et al., 2021	2021	Internet gaming	Internet gaming disorder, social engagement	Adults	Cross-sectional
Yue et al., 2021	2021	Smartphone use	Patient health, social anxiety, short boredom proneness, and smartphone addiction	Adults	Cross-sectional
Hussain et al., 2020	2020	Internet use	Internet addiction, salience, excessive use, neglect of work, anticipation, lack of control, and neglect of social life	Adults	Cross-sectional
Cerniglia et al., 2019	2019	Internet gaming	Internet gaming disorder, social media addiction, impulsiveness, and psychopathology symptom	Adolescents	Cross-sectional
Myrseth and Notelaers, 2018	2018	Gambling and gaming	Disordered gaming, loneliness, depression and anxiety, and aggression	Adults, Adolescents	Longitudinal
Carras et al., 2017	2017	Video gaming	Video game addiction, social internet use, psychosocial well-being, depressive symptoms, loneliness, social anxiety, self-esteem, and friendship quality	Adolescents	Cross-sectional
Faulkner et al., 2015	2015	Video gaming	Problem video game playing, frequency of video gaming, mental health, physical health, physical activity, academic performance, and analytic strategy	Adolescents	Cross-sectional
Wartberg et al., 2015	2015	Internet use	Problematic internet use, family assessment, and life satisfaction	Adolescents	Cross-sectional
Rumpf et al., 2014	2014	Internet use	Internet addiction, social participation and trust	Adults, Adolescents	Cross-sectional
Van Rooij et al., 2011	2011	Video gaming	Compulsive internet use, weekly hours of online gaming, and psychosocial outcome	Adolescents	Longitudinal

This study explored the effects of negative affects on the mechanism of pathological gaming among adolescents by revealing the mediating effects of aggression and self-control based on the S-O-R framework. In particular, pathological gaming mechanisms of the risk group were compared with those of the non-risk group by uncovering latent classes within longitudinal data for adolescent game use. The following research questions were answered. The findings are expected to contribute to bridging the gaps in existing studies.

*RQ1*: How do negative affects work as antecedents of the mechanism of pathological gaming among adolescents in the longitudinal axis?
*RQ2*: How do mediators such as aggression and self-control relate negative affects with pathological gaming among adolescents?
*RQ3*: Does the pathological gaming mechanism of the adolescents in the risk group differ from that in the non-risk group?

## Literature Review and Hypothesis Development

The decision by WHO to include “gaming disorder” in the 11th edition of the International Classification of Diseases (ICD-11) is still under debate. Some scholars supported the decision in that it could drive further research on the subject for better diagnosis and management (Saunders et al., 2017). However, some other scholars have argued that it is premature to officially consider excessive gaming behavior as a mental illness, given that neither reasonable measuring criteria nor sufficient clinical record have been provided (Aarseth et al., 2017; Przybylski et al., 2017). Moreover, “Internet gaming disorder” was not officially viewed as a mental disorder in the 5th version of the Diagnostic and Statistical Manual of Mental Disorders (DSM-5). Therefore, given these arguments, the term “pathological gaming” is controversial. Focusing more on mechanism of gaming behavior in adolescents than on mental disorders, this study uses the term despite controversy.

Pathological gaming is defined as excessive and compulsive use of computer or video games that causes social or emotional problems to the extent that game users cannot control their use of the game (Lemmens et al., 2009). Previous studies have shown that pathological use is manifested by a variety of causal relationships. Unraveling the complex causality between addictive behavior and internal/external causes has been a priority in addiction research. The psychological vulnerability of adolescents in the growing stage will increase the value of identifying the emotional causes of addictive behavior. Furthermore, demonstration of the effects of mediators will refine the relationship between the emotional stimulus and the addictive behavior response. The research model in this study was conceptualized based on the following reviews of literature on negative affects, mediators, and the S-O-R framework in the context of pathological gaming.

### Negative Affects and Pathological Gaming

The cognitive behavioral theory confirms that negative affects evoke distorted cognitive processes, which interfere with individuals’ judgments about problematic behaviors (Caplan, 2002). It points to negative affects such as anxiety and loneliness, along with stress, as factors that instigate the pathological use of media (Caplan, 2003). This study considers how negative affects drive the pathological use of media.

Anxiety is defined as an emotional reaction such as tension, nervousness, worry, or uncertainty that arises from a threatening situation such as a social, physical, or mental crisis (Johnston, 1980). Individuals suffering from anxiety in society immerse themselves in virtual contents in the hope of avoiding interpersonal interaction and conflict (Prizant-Passal et al., 2016). Researchers have found that individuals who experience high levels of anxiety use gameplay as a means to escape from that emotion (Kardefelt-Winther, 2014). A study of pathological gaming among 1,174 students empirically proved that students who were addicted to games had a higher degree of sensitivity to social anxiety than those who were not (Karaca et al., 2020).

Loneliness is assumed to be another significant psychological cause of pathological gaming. It is defined as a negative emotion arising from the subjective perception of poor quality and depth of interpersonal relationships, or lack of interpersonal connections (de Jong Gierveld, 1998). High levels of loneliness lead to health problems such as insufficient sleep and depression, as well as behavioral addictions (de Jong Gierveld, 1998; Jeong et al., 2017; Matthews et al., 2017). Pathological gaming studies suggest that individuals in a lonely state are immersed in online games that facilitate the formation of inter-relational networks in order to compensate for their psychological deficits and to enable them to obtain social rewards. Stress is defined as the degree of perception of one’s life as unpredictable, uncontrollable, and overwhelming (Cohen et al., 1983). Individuals in high-intensity stressful situations prefer short-term rewards and avoidance approaches to problem coping rather than step-by-step solutions (Finset et al., 2002; Brand et al., 2016). Therefore, game users stimulated by severe stress use games excessively as a way to escape from problems in reality (Kwon et al., 2011; Yen et al., 2019). Conversely, recent studies suggested that stress may increase due to pathological gaming. An inter-group comparison study of pathological gaming confirmed relatively low resilience and high stress perception in the addictive game use group (Yen et al., 2019). It shows that health or social problems caused by pathological gaming increase stress (Mei et al., 2016). Thus, while stress triggers pathological gaming, pathological gaming increases stress, and eventually becomes addictive.

### Mediation Roles of Aggression and Self-Control

Examining the mediating effect in the causal relationship of pathological gaming will help fill in the lack of existing research. Research findings that coping with pathological gaming requires controlling and suppressing negative affects (Yen et al., 2018) emphasize the need to explore indirect interventions to achieve direct effects on pathological gaming. The S-O-R model describes the functioning of the mediating role of an individual’s internal or organismal experience in the causal relationship between antecedental stimuli such as negative affects and behavioral response (Arora, 1982). The S-O-R paradigm has recently been expanded to various areas in marketing including to computer or website experience, which can theoretically explain how an individual who is stimulated by the environment, cognition, and emotions becomes immersed in game use through organic reactions such as self-control or aggression. In this study, the term “stimulus” refers to a negative affect such as anxiety, loneliness, or stress, which arouses game users. The term “organism” refers to a mediating variable such as aggression or self-control, which is equivalent to the cognitive, affective, or behavioral mediating state between negative affects and game addiction. The term “response” refers to pathological gaming that shows an individual’s reactive behavior, such as acceptance or avoidance of game use.

The mediating effects of aggression and self-control in the link between negative affects and pathological behavior have been explored. A survey of 789 Koreans found that aggression intervenes in the relationship between depression and pathological gaming (Jeong et al., 2017). Self-control has also been found to mediate between stress and internet addiction (Song and Park, 2019; Mills and Allen, 2020) and to mediate the relationship between stress and smartphone addictive use (Cho et al., 2017). Proof of these is still lacking, however, and little has been studied in the field of adolescent pathological gaming research. Thus, in this study, a research model will be carved from pieces of existing research.

#### Aggression

The organic activation of aggression by negative affects has been clearly confirmed in studies. The recent advancement of virtual interaction has expanded the traditional concept of aggression into a cyber-perspective. In line with the traditional concept of aggression, cyber-aggression can be defined as behavior that attempts to harm others through computers, mobile phones, and other electronic media (Schoffstall and Cohen, 2011).

Individuals with a high level of anxiety are prone to misjudgment of their behavior. To recover from such unreasonable evaluation of their behavior, they tend to solve problems in a radical, revengeful way rather than a long-term, mutually beneficial way (Purdon et al., 2001; Loudin et al., 2003).

Loneliness is also well known as an effective predictor of aggression in early adults (Yavuzer et al., 2019). Individuals who deeply indulge in loneliness perceive their surroundings as threats, so they react aggressively to others to protect themselves (Cacioppo and Cacioppo, 2018). The same goes for stress. The frustration-aggression assumption explains that individual frustration and suffering lead to hostile perceptions and aggressive behavior (Dollard et al., 1939). The hypothesis implies the primitive effect of stress on aggression. In this way, a high level of stress that strongly arouses negative affects can be a serious cause of aggression by interfering with effective coping with and resolution of problems and by favoring hostile approaches such as yelling (Bodenmann et al., 2010).

Coupled with the response step in the S-O-R framework, aggression is considered a major cause of addictive behavior. Aggression seriously exacerbates pathological gaming. For example, there are highly aggressive persons who are excessively engrossed in competitive and violent games in order to consistently produce an aggressive desire (Mehroof and Griffiths, 2010). Since such harsh games often adopt mechanisms that provide rewards for aggressive performance, highly aggressive game users can become more addicted to such games for their needs (Mehroof and Griffiths, 2010).

#### Self-Control

Self-control is a predictor of success in life, psychological health, and subjective happiness (Hofmann et al., 2014). Self-control is defined as the ability of an individual to handle his or her emotions, thoughts, and actions in order to cope with impulses and temptations (Muraven and Baumeister, 2000). Negative affects organically influence self-control.

Anxiety adversely affects self-control by impeding the effectiveness of strategies to persevere and improve certain situations (Cbiederman and Schefft, 1994; Blackhart et al., 2015). Individuals with high anxiety levels have difficulty displaying goal-oriented behavior because they focus on controlling and avoiding anxiety situations (Vohs et al., 2005; Kashdan et al., 2010; Blackhart et al., 2015). Anxiety also decreases self-control functions and reduces the likelihood of control over future events. Particularly, anxiety interferes with the development and maintenance of adolescents’ self-control (Chorpita and Barlow, 1998). Social anxiety is also directly involved in depleting self-control (Kashdan et al., 2013).

Loneliness weakens individuals’ self-control functions by driving them to deal negatively with their emotions (Sinha, 2009; Özdemir et al., 2014). According to the social cognitive theory, psychological problems caused by loneliness or depression can disable self-control by interrupting proper observation or evaluation of the situation (LaRose et al., 2003). High levels of loneliness have been found to be not only correlated with low self-control, but to also have a direct impact on the pathological use of the internet (Davis, 2001; Gámez-Guadix et al., 2012; Özdemir et al., 2014; Tokunaga, 2017).

Coping with stress requires effort to ignore or control negative thoughts or feelings (Oaten and Cheng, 2005). High-stress individuals find themselves in situations, where they lose the cognitive resources needed for self-control by focusing on the reduction or avoidance of negative stimuli (Lin, 1999). Since the self-control of an individual is not infinite, the increase in factors that need to be controlled reduces the effectiveness of self-control (Baumeister et al., 1998; Muraven and Baumeister, 2000). As a result, weakened self-control fails to control problem behavior (Lin, 1999). Therefore, this organic operation shows that stress is negatively related to self-control.

In relation to the response stage of the S-O-R framework, self-control has been considered a key antecedent of addictive behavior (Lee and Shin, 2004; Özdemir et al., 2014). Low self-control is a factor that intensifies addictive behavior. This is because low self-control or high impulse is accompanied by addictive behavior that ignores long-term benefits for immediate satisfaction (Hyun et al., 2015; King et al., 2019). A game user study confirmed that lack of self-control, such as impulsiveness, is a risky cause of pathological gaming, and conversely, self-control can also be weakened by pathological gaming (Chang and Kim, 2020; Jeong et al., 2020).

### Hypothesis Development in the S-O-R Framework

Compared to adults, the relationship of negative affects of adolescents on their pathological gaming have not been sufficiently studied yet. In particular, the mediating effect needs to be more emphasized in the research on adolescent game use because it can elucidate more precisely the mechanism of pathological gaming. The S-O-R framework assumes that the negative affects aroused in adolescents will increase their level of pathological gaming, where aggression and self-control act as mediators. Thus, in this study, the following hypotheses are presented based on the S-O-R framework to analyze the gaming behavior of adolescents in a longitudinal study.

*H1*: The aggression of the adolescents in wave 2 will be stronger with increases in their anxiety (H1a), loneliness (H1b), and academic stress (H1c) in wave 1.

*H2*: The self-control of the adolescents in wave 2 will be weaker with increases in their anxiety (H2a), loneliness (H2b), and academic stress (H2c) in wave 1.

*H3*: The pathological gaming of the adolescents in wave 3 will be stronger with increases in their aggression (H3a) in wave 2, but will be weaker with an increase in their self-control (H3b) in wave 2.

*H4*: The relationship between negative emotions and pathological gaming will be mediated by aggression and self-control.

## Research Methodology

### Data Source and Participants

In this study, data observed for 3 years among longitudinal panel data for the Korean Adolescent Game User Cohort Research were analyzed to track the adolescents before they graduated from secondary school. The panel survey was conducted by the Korea Creative Content Agency (KOCCA) to investigate the game use attitudes of elementary, middle, and high school youth (10˗17 years old). The research was officially approved in advance by the University Ethics Committee, and the informed consent of each participant was obtained with guarantees of privacy protection and anonymity.

Samples were extracted through quota sampling based on school grades and gender ratios. The same questionnaire was answered for each wave at intervals of a year, at which the participants responded to face-to-face interviews by trained professional agents following established survey guidelines. Each panel participant was rewarded with USD27.00. The KOCCA website^1^ provides details on the survey methods and data.

The male and female gender ratios of the 778 subjects in this study were similar (49.0% male and 51.0% female). In the first wave, 36.9% of the subjects were elementary school students, 35.1% were middle school students, and 28.0% were high school students. In terms of their daily gaming time, 360 students were above average (46.3%) and 418 students were average or below average (53.7%). In terms of the game type, 52% of the subjects played mobile games longer than online games, and 47.2% played online games longer than mobile games. Table 2 shows the demographic characteristics of the subjects in both the risk group and the non-risk group.

**Table 2:** Demographic characteristics.

Characteristics		All participants (*n* = 778)		Risk group (*n* = 355)		Non-risk group (*n* = 327)	
		Frequency	(%)	Frequency	(%)	Frequency	(%)
Gender	Male	381	49.0	216	60.8	142	43.4
	Female	397	51.0	139	39.2	185	56.6
Age (in years)	Under 12	287	36.9	143	40.3	109	33.3
	12–14	273	35.1	127	35.8	112	34.3
	15–17	218	28.0	85	23.9	106	32.4
Education	Elementary school	287	36.9	127	35.8	118	36.1
	Middle school	273	35.1	120	33.8	121	37.0
	High school	218	28.0	108	30.5	88	26.9
Online game duration (daily average min.)	Elementary school	38.1	23.7	44.4	23.1	30.0	21.2
	Middle school	62.2	38.8	69.9	36.3	65.2	46.0
	High school	60.2	37.5	78.0	40.6	46.6	32.9
Mobile game duration (daily average min.)	Elementary school	48.4	27.0	54.0	27.6	48.1	27.4
	Middle school	78.2	43.6	80.2	41.0	82.2	46.8
	High school	52.6	29.4	61.5	31.4	45.2	25.8

The demographics above correspond to the first wave.

### Measure Development

The questionnaire items measured three negative affects (anxiety, loneliness, and academic stress) and the levels of the aggression, self-control, and pathological gaming of the adolescent respondents. The questions were adopted from literature and validated by the researchers. Some of them reflected the game context among Korean teenagers. The constructs were measured on the Likert scale.

*Anxiety* was operationally defined as the extent of restlessness, uneasiness, and nervousness experienced by the adolescents in the last 2 weeks. It was assessed with the seven-item Generalised Anxiety Disorder Assessment (GAD-7) questionnaire (Spitzer et al., 2006). The variables were measured on a four-point Likert scale (0 = Never, 1 = Occasionally, 2 = Frequently, and 3 = Always).

*Loneliness* was operationally defined as the degree to which the adolescents felt that they were isolated and separated, lacked friends, had no conversational partners, had meaningless relationships, etc. It was assessed using the 10-item UCLA Loneliness Scale (Version 3; Russell, 1996). The variables were measured on a four-point Likert scale (1 = Strongly disagree, 2 = Slightly disagree, 4 = Somewhat agree, and 5 = Strongly agree).

*Academic stress* was operationally defined as the degree to which the adolescents experienced excessive exam frequency perception, low school achievement, academic difficulties, unfriending, bullying, etc. in school. Academic stress was assessed with seven items related to stress from school life and friendship, which were selected from the Korean version of the Medium School Students Coping Scale for Academic Stress (Kim and Chon, 1993). The variables were measured on a three-point Likert scale (1 = Disagree, 2 = Neutral, and 3 = Agree).

*Aggression* was operationally defined as physical aggression, verbal aggression, anger, and hostility experienced by the adolescents. It was assessed with the revised 12-item Short-Form Buss-Perry Aggression Questionnaire (BPAQ-SF; Diamond et al., 2005) that was a refinement (Bryant and Smith, 2001) of the 29-item self-report BPAQ (Buss and Perry, 1992). This variable was measured on a five-point Likert scale (1 = Strongly disagree, 2 = Disagree, 3 = Neutral, 4 = Agree, and 5 = Strongly agree).

*Self-control* was operationally defined as the adolescents’ ability to handle temptation, bad habits, laziness, careless speech, lack of concentration and patience, etc. It was assessed using the 13-item Brief Self-Control Scale (BSCS; Tangney et al., 2004). This variables was measured on a five-point Likert scale (1 = Strongly disagree, 2 = Disagree, 3 = Neutral, 4 = Agree, and 5 = Strongly agree).

*Pathological gaming* was defined operationally as gaming to the extent that the adolescents perceived problems related to time management and performance, withdrawal and social difficulties, and reality substitutes experienced after immersing in games. Pathological gaming was assessed with Young’s 20-item Scale (Young, 1998) that was adapted and modified for the game context. This variable was measured on a five-point Likert scale (1 = Strongly disagree, 2 = Disagree, 3 = Neutral, 4 = Agree, and 5 = Strongly agree).

### Procedure and Data Analysis

The data were analyzed in three mixed procedures. In the first step, the results were derived from the global research model analysis for the entire sample (*n* = 778) using the partial least squares-structural equation modeling (PLS-SEM) method by analyzing the measurement and structural model that was controlled by the gender, age, and school grade. In the second step, latent groups of pathological gamers were uncovered to compare the results between the risk group and the non-risk group by using the data segmentation technique of finite mixture partial least squares (FIMIX-PLS; Hair et al., 2016). In the last step, the results were compared by applying the procedure in the first step to all three groups in the same way. The SmartPLS software was used for the data analysis (version 3.3.2, Bön-ningstedt, Germany; Ringle et al., 2015).

## Results

### Measurement Model Validation

Using PLS-SEM statistical processing, the measurement model was validated by assessing the statistical criteria of convergent validity (i.e., the factor loading value, AVE), reliability of the internal consistency (i.e., Cronbach’s alpha value, CR), and discriminant validity [i.e., the heterotrait-monotrait (HTMT) ratio]. The PLS-SEM analysis using the SmartPLS software validated the measurement model through acceptable threshold values that complied with the following criteria and requirements (Hair et al., 2017). The outer loadings of individual items with values above 0.7, and the average variance extracted (AVE) with values above 0.5, ensured higher convergent validity. The Cronbach’s alpha and composite reliability (CR) of individual constructs with values above 0.7 ensured higher reliability of the internal consistency. Considering the reliability issue of Fornell and Larcker’s criterion in common research settings (Henseler et al., 2015), this study confirmed discriminant validity using the recent HTMT ratio of the correlations method. The threshold values of less than 0.9 for the HTMT ratio ensured discriminant validity. The results confirmed that the developed measurement model meets the criteria for convergent validity, reliability of internal consistency, and discriminant validity (see Table 3).

**Table 3:** Results for the measurement model.

Scale/Items		Mean	SD	CR	AVE	*R* ^2^	Cronbach’s α
All participants (*n* = 778)	Anxiety (T1)	0.722	0.479	0.916	0.644	-	0.890
	Loneliness (T1)	1.625	0.570	0.926	0.611	-	0.909
	Academic stress (T1)	2.000	0.365	0.874	0.698	-	0.787
	Aggression (T2)	1.952	1.098	0.913	0.600	0.175	0.888
	Self-control (T2)	2.722	0.524	0.884	0.560	0.187	0.843
	Pathological gaming (T3)	3.148	1.042	0.967	0.621	0.246	0.964
Risk group (*n* = 355)	Anxiety (T1)	0.900	0.352	0.903	0.652	-	0.868
	Loneliness (T1)	1.646	0.544	0.925	0.609	-	0.909
	Academic stress (T1)	1.833	0.548	0.867	0.686	-	0.777
	Aggression (T2)	2.310	1.074	0.907	0.585	0.144	0.880
	Self-control (T2)	2.583	0.303	0.862	0.610	0.137	0.788
	Pathological gaming (T3)	3.567	0.557	0.879	0.593	0.153	0.828
Non-risk group (*n* = 327)	Anxiety (T1)	0.250	0.293	0.923	0.667	-	0.900
	Loneliness (T1)	1.571	0.433	0.920	0.622	-	0.899
	Academic stress (T1)	2.056	0.251	0.880	0.709	-	0.797
	Aggression (T2)	1.476	0.380	0.915	0.607	0.181	0.891
	Self-control (T2)	3.083	0.646	0.867	0.620	0.246	0.797
	Pathological gaming (T3)	1.528	0.687	0.876	0.542	0.085	0.832

SD, standard deviation; CR, composite reliability; AVE, average variance extracted; and *R*^2^, R square adjusted. T1, T2, and T3 indicate the periods of wave 1, wave 2, and wave 3, respectively.

### Uncovering Latent Groups of Pathological Gamers

To verify whether the adolescents’ addictive mechanism worked differently between the risk group and the non-risk group, latent classes were previously uncovered based on pathological gaming variables using the FIMIX-PLS method of the SmartPLS software. The method used was a data segmentation technique that discovered unobserved heterogeneity that could not be traced with observable characteristics such as gender, age, or income in the data. This technique is a very advanced LCA approach to PLS-SEM (Hair et al., 2016) that Hahn et al. (2002) first introduced and then Sarstedt et al. (2011) extended.

By following the systematic procedures and guidelines of the FIMIX-PLS method suggested by Hair et al. (2016), a longitudinal LCA model was built by inputting three pathological gaming variables as dependent variables (waves 1–3 measured longitudinally at 6-month intervals) into the authors’ basic research model. In the model, the variables were directly linked to each pathological gaming variable that was controlled by gender and age. This modeling approach is based on a generic model of LCA in longitudinal studies that used covariance-based SEM (e.g., Van Rooij et al., 2011; Myrseth and Notelaers, 2018).

**Figure 1:**
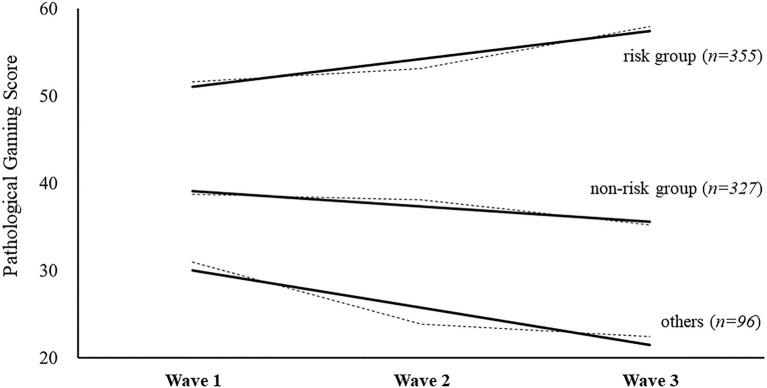
Results of the latent class analysis of pathological gaming. As pathological gaming is measured on a scale of 100 points, the score of 50 is assumed to be the splitting point of the addiction risk tendency. Dotted line connects the measured values of the pathological gaming variables from waves 1–3, and the bold and solid line reflects the trend line.

After running multiple FIMIX-PLS analyses until the relative size of the smallest split group was less than 5% of the original data, the results identified several groups with some fit index outputs in the FIMIX module, such as AIC, AIC3, AIC4, BIC, CAIC, MDL5, and EN. Based on the recommendation of Hair et al. (2016) to select index values for segment determination, the number of segments could be optimally determined. Basically, the optimal solution is the number of segments with the lowest value among the indices. However, in terms of the entropy statistic (EN) index, a higher value indicates better separation of the segments. From the results, three optimal latent groups related to game use patterns among adolescents were identified. The sample sizes of the three groups were 46.3% (*n* = 355), 41.6% (*n* = 327), and 12.1% (*n* = 96), respectively. In the graphical figure of the LCA results (waves 1–3), the largest segmented group was predicted as a risk group (right upward movement slope on the graph) and the second largest group was predicted as a game non-risk group (right downward movement slope on the graph). However, the smallest group (*n* = 96) was excluded from the further analysis due to the insufficient number of samples allowed for PLS-SEM analysis (see Table 4; Figure 1).

**Table 4:** Finite mixture partial least squares (FIMIX-PLS) analysis results for latent class segmentation.

Segment criteria	Prespecified segments						
Fit indices	*K* = 1	*K* = 2	*K* = 3	*K* = 4	*K* = 5	*K* = 6	*K* = 7
AIC	(9,778.851)	8,808.235	8,387.198	8,321.209	8,248.713	8,264.391	8,212.271
AIC3	(9,815.851)	8,883.235	8,500.198	8,472.209	8,437.713	8,491.391	8,477.271
AIC4	(9,852.851)	8,958.235	**8,613.198**	8,623.209	8,626.713	8,718.391	8,742.271
BIC	(9,951.150)	9,157.490	**8,913.408**	9,024.375	9,128.834	9,321.468	9,446.304
CAIC	(9,988.150)	9,232.490	**9,026.408**	9,175.375	9,317.834	9,548.468	9,711.304
MDL5	(10,936.346)	11,154.508	11,922.248	13,045.038	14,161.320	15,365.776	16,502.434
LnL	(−4,852.426)	−4,329.118	−4,080.599	-4,009.605	−3,935.357	−3,905.196	−3,841.136
EN		0.820	**0.840**	0.799	0.767	0.786	0.754
**Number of groups**	**Relative segment sizes (%)**						
	Segment 1	Segment 2	Segment 3	Segment 4	Segment 5	Segment 6	Segment 7
*K* = 1	100%						
*K* = 2	59.5%	40.5%					
***K* = 3**	**46.3%**	**41.6%**	**12.1%**				
*K* = 4	39.3%	35.9%	13.2%	11.6%			
*K* = 5	34.9%	27.0%	19.0%	12.0%	7.1%		
*K* = 6	33.7%	27.3%	12.8%	11.1%	8.9%	6.2%	
*K* = 7	24.5%	20.7%	18.5%	12.8%	12.4%	6.9%	4.3%

The best segment choices are bolded. K indicates the number of prespecified segments; AIC, akaike’s information criterion; AIC3, modified AIC with factor 3; AIC4, modified AIC with factor 4; BIC, bayesian information criteria; CAIC, consistent AIC; MDL5, minimum description length with factor 5; LnL, loglikelihood; and EN, entropy statistic (Normed).

### Hypothesis Test Results

To discern how negative affects and mediators work as antecedents on the pathological gaming mechanism of the adolescents in the longitudinal axis, the proposed hypotheses were tested for these three groups: the entire population group (*n* = 778), the risk group (*n* = 355), and the non-risk group (*n* = 327). The results are briefly summarized as follows.

First, the results of the test of the hypotheses related to aggression (in wave 2) affected by negative affects (in wave 1) are as follows. Anxiety in the three groups had a common statistically significant effect on aggression (*β* = 0.223, *p* < 0.001 in the entire group; *β* = 0.258, *p* < 0.001 in the risk group; and *β* = 0.166, *p* < 0.05 in the non-risk group). Loneliness in the three groups also had a statistically significant effect on aggression (*β* = 0.203, *p* < 0.001 in the entire group; *β* = 0.145, *p* < 0.01 in the risk group; and *β* = 0.284, *p* < 0.001 in the non-risk group). Academic stress had a statistically significant effect on aggression in the entire group (*β* = 0.124, *p* < 0.001) and in the risk group (*β* = 0.119, *p* < 0.05), but not in the non-risk group (*β* = 0.096, *p* > 0.05).

Second, the results of the test of the hypotheses related to self-control (in wave 2) affected by negative affects (in wave 1) are as follows. Anxiety had a statistically significant effect on self-control in the entire group (*β* = −0.107, *p* < 0.01) but not in the risk group (*β* = −0.083, *p* > 0.05) and the non-risk group (*β* = −0.094, *p* > 0.05). Loneliness had a statistically significant effect on self-control in the entire group (*β* = −0.112, *p* < 0.01) and in the non-risk group (*β* = −0.155, *p* < 0.01), but not in the risk group (*β* = −0.065, *p* > 0.05). Academic stress in the three groups had a common statistically significant effect on self-control (*β* = −0.330, *p* < 0.001 in the entire group; *β* = −0.325, *p* < 0.001 in the risk group; and *β* = −0.376, *p* < 0.001 in the non-risk group).

Finally, the results of the test of the hypotheses related to pathological gaming (in wave 3) affected by the three mediating variables in wave 2 (aggression and self-control) are as follows. Aggression in the three groups also had a common statistically significant effect on pathological gaming (*β* = 0.199, *p* < 0.001 in the entire group; *β* = 0.175, *p* < 0.01 in the risk group; and *β* = 0.256, *p* < 0.01 in the non-risk group). However, self-control had a statistically significant effect on pathological gaming in the entire group (*β* = −0.255, *p* < 0.001) and in the risk group (*β* = −0.287, *p* < 0.001), but not in the non-risk group (*β* = −0.079, *p* > 0.05).

**Figure 2:**
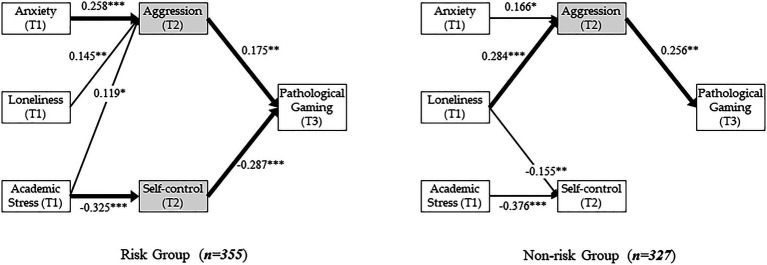
Results of hypothesis test and mediation effect analysis. One asterisk (*) indicates *p* < 0.05, two asterisks (**), *p* < 0.01, and three asterisks (***), *p* < 0.001. Solid line indicates statistical significance. Bold line represents both statistical significance and significant indirect effect pathways. No link is statistically insignificant.

For a brief summary, the common factor in the three groups (the entire population group, the risk group, and the non-risk group) revealed that aggression in wave 2 became stronger with anxiety and loneliness in wave 1, self-control in wave 2 was weakened by academic stress in wave 1, and pathological gaming in wave 3 became stronger with aggression in wave 2. However, the effects of academic stress on aggression, of loneliness on self-control, and of self-control on pathological gaming differed between the risk group and the non-risk group of game addiction. Figure 2 summarizes the hypothesis test results (Table 5).

**Table 5:** Hypothesis test results (direct effect).

Hypothesis	All participants (*n* = 778)			Risk group (*n* = 355)			Non-risk group (*n* = 327)		
	Coef.	T-values	Results	Coef.	T-values	Results	Coef.	T-values	Results
H1a. AX (T1) → AG (T2)	0.223	5.369^***^	Accepted	0.258	4.654^***^	Accepted	0.166	2.375^*^	Accepted
H1b. LL (T1) → AG (T2)	0.203	5.031^***^	Accepted	0.145	2.632^**^	Accepted	0.284	4.374^***^	Accepted
H1c. AS (T1) → AG (T2)	0.124	3.656^***^	Accepted	0.119	2.342^*^	Accepted	0.096	1.848	Rejected
H2a. AX (T1) → SC (T2)	−0.107	2.634^**^	Accepted	−0.083	1.507	Rejected	−0.094	1.632	Rejected
H2b. LL (T1) → SC (T2)	−0.112	3.009^**^	Accepted	−0.065	1.197	Rejected	−0.155	2.959^**^	Accepted
H2c. AS (T1) → SC (T2)	−0.330	10.639^***^	Accepted	−0.325	7.104^***^	Accepted	−0.376	8.404^***^	Accepted
H3a. AG (T2) → PG (T3)	0.199	5.074^***^	Accepted	0.175	3.119^**^	Accepted	0.256	4.088^***^	Accepted
H3b. SC (T2) → PG (T3)	−0.255	6.618^***^	Accepted	−0.287	4.849^***^	Accepted	−0.079	1.315	Rejected

* *p* < 0.05;

** *p* < 0.01 and

*** *p* < 0.001.

AX, anxiety; AG, aggression; SC, self-control; LL, loneliness; AS, academic stress; and PG, pathological gaming.

### Results of the Mediation Analysis

To assess how the mediating variables such as aggression and self-control work in the mechanism of pathological gaming, their specific indirect effects were examined in the SmartPLS analysis results. The method of bootstrapping indirect effects using software such as SmartPLS is perfectly suited to PLS-SEM because it does not make assumptions about the shape of the variables’ distribution, or the sampling distribution of the statistic (Preacher and Hayes, 2008; Hair et al., 2017). In addition, unlike the existing Sobel Test approach (online calculator) or causal procedure approach of Baron and Kenny (1986), this method does not require significant direct effects. This method emphasizes only significant indirect effects, insisting that there is no need for complete mediation or partial mediation in the assumption of mediating effects (Preacher and Hayes, 2008; Hair et al., 2017; Ramayah et al., 2018).

In the results of the mediation analysis of the entire group (*n* = 778), the bootstrapping analysis showed that the seven indirect effects, excluding two paths, are statistically significant as follows: Anxiety → Aggression → Pathological gaming (*β* = 0.044, *t*-value = 3.610, LL = 0.022, and UL = 0.070); Anxiety → Self-control → Pathological gaming (*β* = 0.027, *t*-value = 2.389, LL = 0.007, and UL = 0.052); Loneliness → Aggression → Pathological gaming (*β* = 0.040, *t*-value = 3.335, LL = 0.020, and UL = 0.066); Loneliness → Self-control → Pathological gaming (*β* = 0.029, *t*-value = 2.606, LL = 0.009, and UL = 0.052); Academic stress → Aggression → Pathological gaming (*β* = 0.025, *t*-value = 2.804, LL = 0.010, and UL = 0.046); and Academic stress → Self-control → Pathological gaming (*β* = 0.084, *t*-value = 5.567, LL = 0.057, and UL = 0.116). The indirect effects’ 95% Boot CI Bias Corrected: [Lower Limit (LL), Upper Limit (UL)] did not straddle a 0 in between, which indicates that there was mediation (Preacher and Hayes, 2004, 2008).

However, unlike the entire group, in the risk group and the non-risk group, only two paths are statistically significant. In the risk group (*n* = 355), Anxiety → Aggression → Pathological gaming (*β* = 0.045, *t*-value = 2.651, LL = 0.015, and UL = 0.083) and Academic stress → Self-control → Pathological gaming (*β* = 0.093, *t*-value = 3.784, LL = 0.048, and UL = 0.144) had statistically significant mediation effects. In the non-risk group (*n* = 327), Loneliness → Aggression → Pathological gaming (*β* = 0.073, *t*-value = 2.697, LL = 0.028, and UL = 0.131) had statistically significant mediation effects. The indirect effects’ 95% Boot CI Bias Corrected: (LL, UL) also did not straddle a 0 in between, which indicates that there was mediation.

The results lead to the following critical conclusions. First, in the entire group, it was confirmed that the relationship between negative affects of adolescents and their pathological gaming is mediated by aggression and self-control. Second, the game addiction risk signals in adolescents need to be carefully considered and closely monitored. In the addiction risk group, the relationship between negative affects and pathological gaming was proven to be particularly significantly mediated by aggression and self-control.

## Discussion

This study investigated the effects of adolescent affects on their pathological gaming based on the S-O-R framework, through mediated variables related to game use. A structural equation model was analyzed to see the effects of negative affects of youth such as anxiety, loneliness, and academic stress on the degree of their pathological gaming through the mediation variables such as aggression and self-control, which were closely related to their game use. The focus was on the pathways of the variables through the mediation effects, which were compared between the risk group and the non-risk group through LCA.

First, the results of this study highlighted academic stress as a key factor of the degree of pathological gaming. In the entire group, the initial negative affects of the adolescents (i.e., anxiety, loneliness, and academic stress) were mediated through aggression and self-control, and developed into a high degree of pathological gaming. Among the initial negative affects, academic stress operated as a major factor of pathological gaming by driving both aggression and self-control. In other words, academic stress can lead to a high degree of pathological gaming by lowering the degree of self-control of adolescents or by increasing their degree of aggression on one hand.

Moreover, academic stress was the factor that lowered self-control in the pathway analysis of the mediation effects in the two potential groups (i.e., the risk group and the non-risk group). Looking at the mediation paths of academic stress by group, academic stress increased the degree of pathological gaming in the risk group by weakening self-control. These results show that the main factor that increases pathological gaming among adolescents is academic stress, unlike loneliness and anxiety among adults (Jun and Choi, 2015).

Next, this study showed the key impact of self-control through the differences in the mediation paths between the risk group and the non-risk group. The mediation path of self-control (i.e., Academic stress → Self-control → Pathological gaming) was the most influential result in the risk group, while that of aggression (i.e., Loneliness → Aggression → Pathological gaming) was the strongest result in the non-risk group. These results show that controlling academic stress and self-control in the risk group, while controlling loneliness and aggression in the non-risk group can be an effective mechanism for preventing pathological gaming among adolescents. However, considering that the characteristics of the risk group represent key factors of pathological gaming among adolescents, the results of this study demonstrate that self-control should be regarded as the most crucial factor of pathological gaming in adolescents (Babakr et al., 2019; Chang and Kim, 2020).

The crucial influence of self-control on the variables of the degree of pathological gaming in the risk group was emphasized. In the risk group, self-control had a higher effect than aggression. This result is consistent with that of previous studies that self-control is a protective factor that inhibits a serious state of pathological gaming (Babakr et al., 2019). Weakening of self-control is linked to an increase in impulse (Jeong et al., 2020). Impulsive adolescents are very likely to seek more immediate satisfaction (e.g., Hyun et al., 2015; King et al., 2019). Gaming is an effective medium for providing immediate satisfaction to the youth. Therefore, the more difficult self-control is to practice, the more likely it is to lead to a high degree of pathological gaming through immersion of the youth in game use when stimulated by negative affects.

With regard to aggression, this study showed that aggression mediates between anxiety and loneliness and pathological gaming. In the entire group, both anxiety and loneliness increased aggression and finally increased the degree of pathological gaming, but the path from anxiety (i.e., Anxiety → Aggression → Pathological gaming) was significant in the risk group, and the path from loneliness (Loneliness → Aggression → Pathological gaming) was significant in the non-risk group. The youth’s immersion in games was also due to their interest in the competitive mechanisms of the games (Rapp, 2021). In other words, the desire to use violent games is likely to increase as a way to spew out the increased aggression among adolescents due to anxiety and loneliness (Mehroof and Griffiths, 2010). In addition, the use of violent games may act as a stress alternative for adolescents. That is, when they are stimulated by negative affects, they will experience stress relief by expressing their aggressive feelings through violent game use, and through further immersion in games, they could reach a serious state of pathological gaming.

Regarding negative affects, this study showed their direct effects on aggression and self-control. In the entire group, negative affects (anxiety, loneliness, and academic stress) all significantly increased aggression and lowered self-control. These results are consistent with most outcomes of previous studies on game users (Cbiederman and Schefft, 1994; Qualter and Munn, 2002; LaRose et al., 2003; Loudin et al., 2003; Bodenmann et al., 2010). Furthermore, the results of this study provide empirical evidence of the comprehensive mechanism of the effects of negative affects on pathological gaming through the mediation paths of aggression and self-control, which has been reported partially in previous studies.

On the other hand, a high degree of academic stress could add to anxiety. Anxiety has been described as a significant factor of adolescent aggression, in which context the perceived academic stress of youth causes anxiety, which will again cause youth aggression (Felson, 1992). Stressed people focus on either relieving their problems or avoiding the pressure to relieve their problems. Such efforts consume too much cognitive energy (Lin, 1999). The pressure to achieve in academics, including through high grades, is a strong source of stress for the youth. Therefore, as adolescents who are exposed to academic stress situations focus too much on relieving such stress, self-control of their negative thoughts and feelings will become difficult.

This study makes several contributions to gaming research. First, through LCA that considers the core characteristics of adolescent gamers, the risk group was distinguished from the non-risk group. Few studies of pathological gaming among adolescents have used potential class analysis. In addition, through the use of the structural equation model for the risk group, this study manifested core factors of adolescent game use such as self-control and academic stress. Moreover, the mediated effects between negative affects and pathological gaming were examined. The analysis of mediation effects highlighted the core paths to pathological gaming such as the self-control path in the risk group. Finally, longitudinal data were used in this study. Through the analysis of long-term data, much clearer mechanisms of pathological gaming among adolescents were revealed.

The results of this study also have practical implications for social policies and activities. First, social understanding of the mechanism of pathological gaming among adolescents is likely to contribute to the spread. Traditionally, gaming has been seen as having a potential risk of pathological use. For example, some scholars argue that digital games are at risk of pathological use in as much as they are subject to user immersion, which implies why it is important to intervene in as early as the structural mechanism design phase in game design (Griffiths and Pontes, 2020). In accordance with this view, a ban on late-night youth gaming, such as through the shutdown system, has been carried out, despite the controversy over its effectivity. However, in this study, the key variable that led to pathological gaming among the adolescents was academic stress, and the variable that most strongly mediated between academic stress and pathological gaming in the risk group was self-control. This suggests that preventing and suppressing pathological gaming among adolescents require much attention to mental health care and self-control in adolescent game use.

This study therefore implies that to properly understand the phenomenon of pathological gaming among adolescents, the cultural context and the social structure must be fully considered. The findings of this study highlight the importance of academic stress, which weakened self-control in the risk group. In a cultural context such as that of Korea, where there are high social expectations of academic achievement, simply suppressing the use of games by adolescents is not a fundamental alternative. To reduce the academic stress of the youth, they may be provided alternative forms of leisure, or else a new consensus may be drawn on excessive pressures toward academic achievement.

Next, expanding the number of counseling or treatment agencies that can manage mental health problems of adolescents will likely help alleviate pathological gaming problems among adolescents. The results of this study showed that negative affects of adolescents and their self-control and aggression are closely related to their pathological gaming. Previous studies have also shown that institutions need to have a combination of self-control over gaming time and suppression programs, as well as programs that can help the adolescents understand their negative affects and strengthen their self-control (Jeong et al., 2017).

This study had the following limitations, however. First, anxiety, loneliness, and stress are not the only affects that may be related to pathological gaming among adolescents. Adolescents can experience other negative affects such as fear, disgust, and anger, besides positive affects such as satisfaction and pleasure, in their developmental stage. Therefore, future research could consider these other affects. Second, the mechanism of pathological gaming among adolescents was explained only with the variables of aggression and self-control. However, adolescents can be exposed to other stimuli in their relationships with their parents and teachers, Thus, future studies could use additional analytical methods such as those that analyze moderated mediation effects on additional variables to understand mediation effects better.

## Conclusion

This study empirically confirmed that negative affects in adolescents are significant factors of the degree of their pathological gaming. Specifically, based on the S-O-R mechanism, this study demonstrated that negative affects in adolescents increase the level of pathological gaming through aggression and self-control. Academic stress played a key role in the entire group. Self-control mediated the effect of academic stress on pathological gaming in the risk group, and showed stronger direct effects than aggression. Considering the increasing social concerns over the risk group, self-control and academic stress should be paid much attention in fundamentally controlling pathological gaming among adolescents. These findings are expected to contribute to the development of research and social policies for adolescent game users.

## Data Availability Statement

The data used in this study are available with permission from Korea Creative Content Agency (KOCCA, http://www.kocca.kr/gameguide/contents.do?menuNo=203709, accessed on Jun. 23, 2021).

## Ethics Statement

The studies involving human participants were reviewed and approved by Konkuk University. Written informed consent to participate in this study was provided by the participants’ legal guardian/next of kin.

## Author Contributions

HJ conducted original draft preparation, data processing and analysis. EJ supervised, performed discussion section, and reviewed the manuscripts. SL performed literature review, data analysis and discussion section. JK performed literature review and editing. All authors contributed to the article and approved the submitted version.

## Conflict of Interest

The authors declare that the research was conducted in the absence of any commercial or financial relationships that could be construed as a potential conflict of interest.

## Publisher’s Note

All claims expressed in this article are solely those of the authors and do not necessarily represent those of their affiliated organizations, or those of the publisher, the editors and the reviewers. Any product that may be evaluated in this article, or claim that may be made by its manufacturer, is not guaranteed or endorsed by the publisher.

## Footnotes

^1^ www.kocca.kr
